# Comparing the Coronal Flaring Efficacy of Five Different Instruments Using Cone-Beam Computed Tomography

**DOI:** 10.7508/iej.2015.04.011

**Published:** 2015

**Authors:** Amin Homayoon, Mahmood Reza Hamidi, Azam Haddadi, Zahra Sadat Madani, Ehsan Moudi, Ali Bijani

**Affiliations:** a * Endodontist, Private Practic, Babol, Iran; *; b * Department of Endodontics, Dental School, Babol University of Medical Sciences, Babol, Iran;*; c * Department of Endodontics, Dental school, Mazandaran University of Medical Sciences, Sari, Iran; *; d * Department of Oral and Maxillofacial Radiology, Dental School, Babol University of Medical Sciences, Babol, Iran; *; e * Non-Communicable Pediatric Diseases Research Center, Babol University of Medical Sciences, Babol, Iran*

**Keywords:** Cone-Beam Computed Tomography, Coronal Flaring, Coronal Pre-Enlargement, Root Canal Treatment, Root Canal Preparation

## Abstract

**Introduction:**

Fearless removal of tooth structure during canal preparation and shaping has negative effects on the prognosis of treatment. On the other hand, sufficient pre-enlargement facilitates exact measurement of the apical size. The present *in vitro *study aimed to compare the efficacy of Gates-Glidden drills, K3, ProTaper, FlexMaster and RaCe instruments in dentin removal during coronal flaring using cone-beam computed tomography (CBCT).

**Methods and Materials:**

A total of 40 mandibular molars were selected and the coronal areas of their mesiobuccal and mesiolingual root canals were randomly prepared with either mentioned instruments. Pre- and post-instrumentation CBCT images were taken and the thickness of canal walls was measured in 1.5- and 3-mm distances from the furcation area. Data were analyzed using the one-way ANOVA. Tukey’s post hoc tests were used for two-by-two comparisons.

**Results:**

At 1.5-mm distance, there was no significant difference between different instruments. However, at 3-mm distances, Gates-Glidden drills removed significantly more dentin compared to FlexMaster files (mean=0.18 mm) (*P*<0.02); however, two-by-two comparisons did not reveal any significant differences between the other groups.

**Conclusion:**

All tested instruments can be effectively used in clinical settings for coronal pre-enlargement.

## Introduction

Increasing use of engine-driven instruments during root canal preparation, necessitates a correct understanding of their properties and limitations. NiTi instruments are superior to stainless steel files due to the superelastic properties. This characteristic make endodontic files more elastic and increase their compliance with the root curvature and their resistance against fracture [1]. 

The aim of cervical preparation is to gain direct access to the apical area of the canals or the apical curvatures [2]. The cervical third of the canals should be prepared safley and the homogeneity of root canal walls should be preserved without the risk of perforation or creation of thin root canal walls [3]. On the other hand, sufficient coronal pre-enlargement can determine the size of initial apical instrument [4-6]. In addition, this technique can be used for more accurate estimation of the root canal working length.

The mesial roots of mandibular molars and the mesiobuccal roots of maxillary molars (*aka* the danger zone) have thinner distal walls which might be endangered during the use of different instruments for endodontic treatment. Recently, Mahran and AboEl-Fotouh [7] reported that ProTaper files removed less dentin from the cervical area of the distal canal walls compared to Hero Shaper and Gates-Glidden drills. However, Sanfelice *et al*. [8] evaluated the efficacy of different instruments, including Gates-Glidden, ProTaper, K3 and LA Axxess instruments, with the Cone-Beam Computed Tomography (CBCT) technique and did not report any significant differences between the groups regarding the amount of dentin removal. 

As the final phase of manual preparation of the canal, flaring is usually carried out with hand stainless steel instruments. This phase is particularly the most difficult part of root canal treatment for general practitioners, during which serious errors occur, resulting in treatment failure, usually through ledge formation, canal transportation or stripping [9, 10]. 

FlexMaster files (VDW, Munich, Germany) have a triangular cross-section with K-type cutting blades and no radial lands. Different tip sizes are also available with 2, 4 and 6% tapers. IntroFile (20/0.11) is the primary orifice shaper with 11% taper and a 9-mm cutting blade. ProTaper system (Dentsply Maillefer, Ballaigues, Switzerland) is amongst the pioneer engine driven instruments with full 360^°^ rotation with a convex triangular cross-section and an advanced flute design that combines multiple tapers within the shaft. The original basic system is comprised of six instruments including three shaping files (SX, S1 and S2) and three finishing files (F1, F2 and F3) [2-4].

RaCe instruments (Reamer with Alternating Cutting Edges) (FKG Dentaire, La-Chaux-de Fonds Switzerland) have a triangular cross-sectional design with alternative cutting edge which is aimed at reducing the tendency to thread the file into the root canal. The exception is the 20/0.02 files, which have a square cross-section. RaCe is marketed with variable tapers (2, 4, 6, 8 and 10%) [5, 11, 12]. 

K3 instruments (Sybron Endo, Orange, CA, USA) have K3 instruments (Sybron Endo, Orange, CA, USA) have a slightly positive rake angel for greater cutting efficiency, wide radial land (which makes the instrument more resistant to torsional/rotational stresses) and feature a *radial land relief*, which aids in protecting the file from over engagement in the canal. The K3 system also offers a third radial land to help prevent threading. The instruments are available with 12, 10, 8, 6 and 4% tapers [11-16].

CBCT is an imaging system which provides three-dimensional (3D) scans from the maxillofacial skeleton; it has overcome the limitations of intraoral radiographic techniques. Compared to the conventional intraoral radiographic techniques, CBCT is more effective in collecting sufficient information for the diagnosis and achieving more capabilities in the management of complicated problems of endodontics [14, 17]. 

The aim of the present *in vitro *study was to compare the efficacy of all aforementioned systems (*i.e.* ProTaper, RaCe, K3, FlexMaster and Gates-Glidden burs) in coronal pre-enlargement of root canals, using CBCT.

## Materials and Methods

The research protocol was approved by the Ethics committee of Babol University of Medical Sciences, Babol, Iran and was conducted on 40 extracted mandibular first molars. The teeth had no restorations and had been extracted due to extensive destruction of coronal structures or periodontal problems. The teeth were kept in 0.1% thymol solution at 9^°^C for disinfection. The teeth were washed with running tap water 24 h before use, to eliminate traces of thymol and were then stored in normal saline at 4^°^C until further processing. Assessment radiographic images were taken using E-speed films (AGFA, Heraeus Kulzer GmbH; Hanau, Germany) with 70 kVp and 8 mA; the films were processed by a Hope film processor. The exclusion criteria included a more-than-3 mm distance between the CEJ and furcation area on radiographs, previous endodontic treatment, incomplete root formation, signs of internal root calcification, external or internal root resorption and more than 40^° ^root curvature (according to Schneider’s method [10]). All the eligible teeth were mounted in dental stone. The samples underwent a pre-instrumentation CBCT imaging using NewTom VG 9000 CBCT device (Quantitative Radiology SRL Co., Verona, Italy) with 80 kVp, 10 mA and 20 sec time and FOV=16×18 cm. Then 0.5-mm axial cross-sections were obtained at 1-mm distances. The radiographs were magnified 4 times using the NTT Viewer software program (NTT Software Corporation, Yokohama, Japan). Then a line was drawn from the mid-buccolingual zone in the distal wall of the canals perpendicular to the external surface of the root. The distance from the distal wall of the mesiobuccal and mesiolingual canals to the distal surface of the mesial root of each tooth was measured in 1.5 and 3 mm distances from furcation zone towards the apex.

The working length (WL) was determined for preparation of the canals. A #10 K file (Mani, Tochigi, Japan) was placed in the root canal so that its tip was visible at the apical foramen; the WL was set 1 mm short of the file length. The teeth were randomly divided into 5 groups including 8 mesiobuccal and 8 mesiolingual root canals in each group. The groups were instrumented as follows: *Group 1* (Gates-Glidden drills): The root canals were prepared using #3, 2 and 1 Gates-Glidden drills (Dentsply, Maillefer, Switzerland) installed on a low-speed handpiece operating at 12.000 rpm. The drills were used directionally in an anti-curvature mode to selectively remove dentin from the bulky wall (safety zone) toward the line angle, protecting the danger zone; *Group 2* (K3): In this group, 25/0.12, 25/0.10 and 25/0.08 files were used with a gear reduction handpiece powered by a on an electric motor (Endo-Mate TC, NSK, Nakanishi Inc., Tokyo, Japan) set at a speed of 300 rpm and torque of 2 Nm; *Group 3* (ProTaper): Root canal preparation was carried out with SX, S1 and S2 instruments set on the same device with speed and torque of 300 rpm and 3 Nm, respectively; *Group 4* (FlexMaster): IntroFile (20/0.11) was used for coronal pre-enlargement with a speed of 300 rpm; *Group 5. *(RaCe): In this group, 40/0.10 and 35/0.35 files were used with a speed of 600 rpm and the torque was set at 1.5 Nm. 

The root canals were irrigated with 2 mL of 2.5% NaOCl between instruments. After preparation, the root canals were irrigated with saline and 2 mL of 2.5% NaOCl to remove all dentin debris. Each file series was discarded after use in one canal or when any defect or deformation was observed in the file. 

Debridement was carried out by one operator. The operator debrided the canals at a specific time of the day and worked only on 5 canals each day so that a constant and uniform force would be applied during canal preparation and the operator fatigue would not exert any effect on the results. Then the samples were placed in the CBCT unit and a post-instrumentation image was taken in the same manner. Then, 0.5-mm axial cross-sections were prepared at 1-mm intervals. The mean values of dentine removal, standard deviations, mean standard errors and 95% confidence intervals of interval differences of the amounts of dentin removed were calculated before and after preparation with different instruments and the data was analyzed with one-way ANOVA. Due to the presence of statistical significance at 3-mm cross-sections, Tukey’s post hoc test was used for two-by-two comparisons of instruments in relation to the amount of dentin removal.

## Results

The mean±SD of the dentin removal value at 1.5- and 3-mm distances from the furcation, were 0.280±0.22 and 0.278±0.22 mm, respectively with no statistically significant differences (*P*=0.93). 

Table 1 represents the central distribution parameters of dentin removal at the first and second cross-sections with different instruments. At 1.5-mm sections and with the use of Gates-Glidden drills, K3, ProTaper, FlexMaster and RaCe, the amounts of dentin removal were 0.243±0.2250, 0.3187±0.1223, 0.3187±0.1721, 0.1563±0.2250 and 0.3625±0.802 mm, respectively. In addition, at 3-mm sections, the amounts of dentin removal were 0.4312±0.2676, 0.2500±0.2098, 0.2437±0.1632, 0.1875±0.1784 and 0.2750±0.2324, respectively. One-way ANOVA showed no significant differences in dentin removal in 1.5 mm sections among different instruments (*P*=0.06); however, the differences at 3-mm cross-sections were significant (*P*<0.025). 

Two-by-two comparisons of instruments at 3-mm sections showed that differences in dentin removal were only significant between FlexMaster and Gates-Glidden instruments (*P*<0.02); however, the differences between other groups were not significant. At 1.5-mm sections, two-by-two comparisons were not made because one-way ANOVA did not reveal any significant differences among the test groups.

**Table 1:** Dentin removal in different groups at 1.5 and 3 mm sections

**Instruments**	**1.5** **mm**	**3 mm**
	**Mean (SD) **	**Mean (SD)**
**Gates-Glidden**	0.24 (0.56)	0.43 (0.06)
**K3**	0.31 (0.03)	0.25 (0.52)
**ProTaper**	0.31 (0.04)	0.24 (0.04)
**FlexMaster**	0.15 (0.05)	0.18 (0.04)
**RaCe**	0.36 (0.07)	0.27 (0.05)
***P*** **-Value **	0.056	

## Discussion

This *in vitro* study compared the coronal-enlargement efficacy of different endodontic instruments using CBCT. Nowadays use of CBCT imaging technique has gained attention due to easy access to processing programs such as Photoshop [15]. This technique has been used to determine the amount of dentin removal during root canal preparation and shaping [8] which is more accurate than routine radiographic techniques. It does not require destruction of samples, it is highly reproducible, provides several images from the root canals and provides detailed information about the root canal before, during and after mechanical preparation [16-18]. In addition, it is possible to use the technique with small equipment and low costs [19]. Hartman *et al.* [15], showed that CBCT technique is reproducible and does not require destructive sectioning of samples or loss of intra-canal materials during root sectioning. Moreover, CBCT can be used as an appropriate tool to identify the initial internal morphology of teeth [20]. In previous studies, techniques such as plastic models [21], histologic cross-sections [22], electron microscopes [23], serial sectioning [24] and radiographic comparisons have been used to evaluate the results of root canal preparation. Mahran *et al.* [7], used multi-slice computed tomography as a practical non-destructive technique to determine the thickness of cervical dentin after using different kinds of burs. At the same time, Sanfelice *et al.* [8] used CBCT technique to determine the amount of dentin removal with the use of different root canal preparation systems, similar to the present study. 

CBCT technique was used in the present study to evaluate the samples due to the advantages mentioned above. There were no significant differences between different root canal preparation systems and instruments in the amount of dentin removal in 1.5-mm cross-sections. However, at 3-mm apical to the furcation, Gates-Glidden drills removed significantly more dentin compared to FlexMaster files. Other two-by-two comparisons did not reveal any significant differences among different systems.

Sanfelice *et al.* [8] did not report any significant differences in dentin removal between Gates-Glidden, ProTaper, K3 and LA Axxess burs, which is somewhat consistent with the results of the present study [8]. However, the results reported by Mahran *et al*. [7] were different and less dentin was removed with the use of ProTaper files, compared to the use of #3 Gates-Glidden drills, from the distal walls of mesiobuccal canals; however, the total amount of dentin removed by the ProTaper system was higher. Gates-Glidden drills are almost inflexible, which is important regarding the narrowing of furcation areas and creation of critical dentin thicknesses in the cervical areas [25-27]. Based on the results of a study by Estrela *et al.* [27], in inexperienced hands or when the path of insertion is not correct, Gates-Glidden drills might result in stripping.

In the study by Flores *et al.*[28], no differences were reported between #2 and 3 Gates-Glidden drills, #1 and 2 Largo burs, #1 and 2 LA-Axxess burs and CP drill (1-size only) on the residual dentin thickness.

In another study by Kássio *et al.* [29], no differences were observed between Gates-Glidden and TripleGates burs and both instruments were safe for cervical preparation .

Marco *et al.* [30] compared Gates Glidden, LA Axxess burs and OrificeShaper instruments, regarding dentin thickness and reported no difference. They concluded that LA 35/0.06 and #3 Gates Glidden drills produced the thinnest dentin walls, and thus their use in mesial canals of mandibular molars should be considered with caution.

In the study by Sanfelice *et al.* [8], where no differences were reported between #1 and 2 Gates-Glidden drills (0.5 and 0.7 mm diameters, respectively) and other systems regarding the amount of dentin removal, the drills were used towards the mesial wall (anti-curvature instrumentation), which resulted in no differences between Gates‒Glidden and other groups. In the present study, #1, 2 and 3 Gates-Glidden drills were used with anti-curvature movements during all the preparation procedures. 

## Conclusion

All the tested instruments had similar efficacy in coronal pre-enlargement and are safe enough for clinical use.
